# Bridging early life trauma to difficult-to-treat depression: scoping review

**DOI:** 10.1192/bjb.2024.75

**Published:** 2025-12

**Authors:** Walter Paganin, Sabrina Signorini, Antonio Sciarretta

**Affiliations:** 1 University of Tor Vergata, Rome, Italy; 2 StudioPsicologiaSignorini, Rome, Italy; 3 IHG, Rome, Italy

**Keywords:** Difficult-to-treat depression, early life trauma, treatment-resistant depression, comorbidity, scoping review

## Abstract

**Aims and method:**

Accumulating evidence suggests that early life trauma (ELT) initiates and perpetuates a cycle of depression, leading to challenges in management and achieving remission. This scoping review aimed to examine the intricate relationship between ELT and difficult-to-treat depression (DTD). An extensive literature search from 1 January 2013 to 21 October 2023 was conducted using the Cochrane Library, PubMed, Scopus, PsycINFO and OpenGrey.

**Results:**

Our review identified scientific literature illustrating the multifaceted link between ELT and DTD, highlighting the dual impact of ELT on therapeutic resistance and clinical complexity.

**Clinical implications:**

This complexity hampers management of patients with DTD, who are characterised by limited pharmacological responsiveness and heightened relapse risk. While exploring the ELT–DTD nexus, the review revealed a paucity of literature on the impact of ELT within DTD. Findings underscore the profound link between ELT and DTD, which is essential for comprehensive understanding and effective management. Tailoring treatments to address ELT could enhance therapeutic outcomes for patients with DTD. Future studies should use larger samples and well-defined diagnostic criteria and explore varied therapeutic approaches.

## Depression: beyond sadness

Depression, in its broadest sense, is a psychological condition marked by feelings of sadness and lack of interest in life's activities.^1^ Although it is common for individuals to experience transient periods of sadness or ‘feeling down’, these emotions are generally short-lived and can be related to specific events or circumstances. When these feelings become pervasive and persistent and begin to interfere with daily functioning, the progression from a mere emotional state to the clinical condition of depressive disorder occurs. This is a syndrome involving significant disturbances in emotions, neurocognitive functions, physiological functions and behaviour, indicating dysfunctions in psychological, biological or developmental processes, according to the ICD-11^2^ and DSM-5 TR.^3^ Depressive disorder is associated with considerable disability and dysfunction in the personal, emotional, family, work and social realms, necessitating clinical intervention and treatment when these exceed the normal variations of behaviour from an individual, social or cultural perspective.^4^ Biological factors such as imbalances in neurotransmitters or hormones and genetic predispositions, as well as external factors such as trauma, loss and prolonged stress, contribute to the transition from simple states of sadness to various depressive disorders.^5^ In the ICD-11, developed by the World Health Organization with a global clinical perspective, depressive disorders are grouped into several categories: single-episode depressive disorder, recurrent depressive disorder, dysthymic disorder, mixed depressive and anxiety disorder, premenstrual dysphoric disorder, other specified depressive disorders, and unspecified depressive disorders. In the DSM-5, developed by the American Psychiatric Association and dominated by an Anglophone-American perspective, depressive disorders are classified as follows: disruptive mood dysregulation disorder, premenstrual dysphoric disorder, persistent depressive disorder (dysthymia), major depressive disorder, substance/medication-induced depressive disorder, and depressive disorder due to another medical condition. Research has shown that the origin of depression lies in a complex interplay of genetic and environmental factors. A family history of depression increases the risk of developing the condition,^6,7^ and factors including neuroinflammation, neurodegenerative diseases, daily stress and traumatic experiences are particularly significant in older adults.^8–10^ Adverse childhood experiences (ACEs), including early traumas, have been recognised in terms of their impact on both post-traumatic stress disorder (PTSD) and depression.^11–13^ The interaction between genetic predisposition and environmental factors, particularly early life trauma (ELT), plays a fundamental part in the development of depressive disorders that are difficult to treat, making their management a complex challenge.

## Approaches to difficult-to-treat depression

**Fig. 1:**
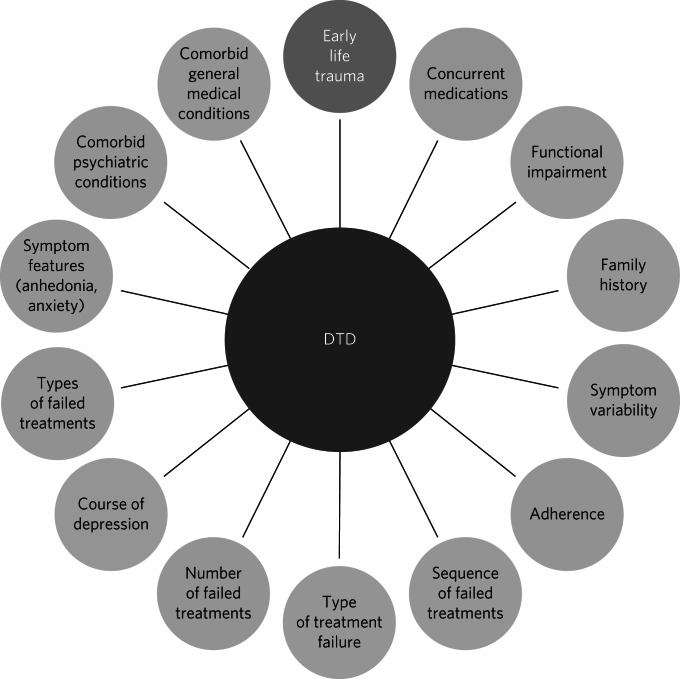
Key factors associated with difficult-to-treat depression (DTD), including early life trauma.

In the past, the term ‘difficult-to-treat depression’ (DTD) was used interchangeably with ‘treatment-resistant depression’ (TRD). However, a significant paradigm shift occurred in 2020 when an international consensus recommended expanding the TRD model to encompass a more comprehensive perspective.^14^ The TRD model that emerged from studies by Thase and Rush^15^ was limited to individuals who showed resistance only to antidepressant medications or those who could not tolerate the initial drug. Thase and Rush proposed a five-stage model to address resistance to different classes of antidepressant. Over time, this model has been replaced by other classifications and has evolved and been variably defined to describe the failure of antidepressant pharmacological treatment in general. A systematic review identified 155 different definitions of TRD,^16^ whereas Wijeratne and Sachdev^17^ noted that there are no formal diagnostic criteria for TRD, and one of its limitations is its exclusive application to acute treatments without considering the possibility of relapse after a transitional period of remission. Subsequently, Rush et al^18^ listed a series of considerations regarding the concept of TRD. They questioned whether a lack of response or remission could define a treatment as failed, how to consider cases where patients improve but then relapse, whether to include cases where a medication cannot be tolerated in the definition of TRD, and whether previously failed treatments should be considered. Furthermore, they noted that definitions of TRD generally do not include non-pharmacological treatments, such as psychotherapy and psychosocial interventions, stating that ‘TRD has no practical, actionable clinical implications other than to suggest attempting another, primarily pharmacological, treatment trial with a different intervention or combination’.^18^ Currently, clinicians and researchers tend to adopt the definition formulated at the turn of the millennium by Souery et al^19^ and later adopted by the US Food and Drug Administration and the European Medicines Agency which defines TRD as the failure to respond to two or more antidepressant pharmacological regimens despite adequate dose and duration, as well as treatment adherence. In recent years, a significant transition from the TRD model to the DTD model has occurred. The DTD model highlights the importance of a perspective that transcends resistance to pharmacological treatments, favouring a broader and more inclusive approach to treating depression. It underscores the need to consider psychological, social and environmental factors that may influence the response to therapy. DTD is not conceived as a binary condition of therapeutic responses but rather as a continuum that includes full responses, partial responses and non-responses, thus shifting the focus of treatment from a curative model of symptom remission to a disease management model that emphasises functional improvement and quality of life, aiming for optimal symptom control without necessarily achieving complete elimination.^20^ Currently, there are no uniform operational criteria used to precisely and measurably define DTD. However, the international consensus statement^14^ proposed adopting a list of factors related to treatment, symptomatology and the patient to identify a clinical framework for DTD. A series of interventions were indicated, ranging from self-help methodologies to psychotherapies, pharmacological strategies, neurostimulation techniques and social interventions to support and facilitate access to general and specialised care and job integration. This will require an individualised intervention strategy that emphasises not only symptom remission but also improvement of the patient's psychosocial functionality. Whereas some patients may show a partial response to initial treatments yet remain functionally impaired, others may show no response. The clinical framework of DTD perceives the condition as treatable (albeit challenging) and underscores the need for personalised care and overcoming routine therapeutic paradigms. DTD encompasses a spectrum of severity and functional impairment, often coexisting with psychiatric or somatic comorbidities. Characterising DTD in clinical terms remains challenging and requires a sophisticated and multidimensional understanding.^21^ This involves considering various aspects, including clinical manifestations, disease progression, biomedical elements, prognostic markers, neuropsychological aspects and treatment responses. Critical factors include the extent and duration of functional decline, symptom history, relapse patterns and the initial clinical profile, particularly the nature and number of unsuccessful therapeutic interventions, psychiatric and organic pathologies, and a history of childhood trauma. A prevalent observation among patients with TRD is a decline in the effectiveness of previously effective pharmacological treatments, suggesting changes in crucial neurobiological foundations. This observation also raises a concerning possibility: ineffective or partially effective treatments may induce neurobiological changes, reducing treatment responsiveness and exacerbating the intractability of depression.^22–24^ Treatment resistance can worsen, leading to increased chronicity. Unlike a single episode of depression that typically responds to treatment and has no lasting impact on personality, persistent depression, with or without appropriate intervention, can shape the clinical trajectory of DTD. Patients with DTD may develop maladaptive behavioural and cognitive patterns that intensify pessimism and negative self-perception. Essentially, depression perpetuates itself, amplifying self-destructive thought patterns and worsening the condition. In evaluating patients with DTD, it is important to consider the clinical presentation of depressive symptoms, with a specific focus on the incidence of anhedonia and anxiety, as well as psychiatric comorbidities and/or concurrent general medical conditions, including substance use disorders. Furthermore, a meticulous evaluation of pharmacological interventions and the patient's functional deterioration is warranted.^20^ The DTD model proposes that in cases where the patient with depression remains unresponsive to treatment, it is important to identify the underlying causes. In the context of medical history, attention should be paid to the number and sequence of previous therapeutic attempts, the types and number of therapeutic failures, as well as family history and treatment adherence. In addition, it is particularly important to identify significant childhood emotional traumas.^11,25^ Events that may occur during an episode of major depression and can lead to DTD include: poor acute response to initial pharmacological treatment, risk of relapse despite ongoing treatment, and the use of multiple antidepressants. These factors could be used to help identify affected patients and to recognise subgroups or spectra of patients, potentially together with use of neuroinflammation biomarkers. A specialised taxonomy for DTD could be developed on the basis of assessment of distinctive characteristics that separate DTD cases from non-DTD cases. Such considerations may include comorbid general medical conditions, the nature and severity of anxiety symptoms, the presence of anhedonia, the extent and chronicity of environmental stressors, concurrent substance use or misuse disorders, and a history of early trauma or abuse;^14,26^ these factors may interact with other characteristics of DTD, adding complexity to the presentation. Psychosocial determinants of health may act as perpetuating factors and thus warrant attention to improve treatment outcomes. These determinants can include chronic stressors related to occupation, marital status, financial conditions or health problems, as well as lifestyle-related factors such as sedentary behaviour and obesity (see Fig. 1 for a summary of these characteristics).

Moreover, predisposing elements such as ELT may have persistent effects on DTD by compromising resilience and problem-solving abilities.^11^ In patients with DTD, ELT events represent important risk factors that can exacerbate the condition or develop into comorbid disorders, further complicating clinical treatment of depression.^18^ A history of ELT can also influence the effectiveness of therapeutic approaches, potentially compromising treatment outcomes. A comprehensive assessment focusing on potential ELT and the possibility of medical and psychiatric complications is crucial to devising effective therapeutic strategies for patients with DTD.

## Definition of ELT

The term ACE was introduced by Vincent Felitti and Robert Anda between 1995 and 1997 to describe various forms of childhood trauma with significant long-term effects on physical and mental health. Initially, ACEs encompassed seven domains: physical, sexual and emotional abuse; living with individuals experiencing mental illness or substance misuse; and having an incarcerated family member.^27^ Over time, the definition expanded to include parental separation, neglect and societal factors such as poverty, community violence, homelessness and intra-community violence. The concepts of early life stress (ELS) and ACEs encompass a wide array of stress-inducing and negative events. These events include but are not limited to maltreatment, neglect, parental separation or loss, exposure to extreme poverty, starvation, and various forms of violence within domestic, community or educational environments. Such experiences can occur at any point from infancy and throughout adolescence. ELS and ACE thus serve as umbrella terms, covering a diverse range of stressful or adverse experiences. Conversely, ELT and childhood trauma have been defined with greater specificity, referring to events that inflict significant traumatic impact. These classifications are reserved for events that pose imminent risks of harm or death or significantly compromise the physical safety of the individual or others. Such traumatic events trigger profound responses of horror, fear or helplessness, overwhelming an individual's coping mechanisms. Examples include acts of terrorism, experiences of war, natural and human-made disasters, physical and sexual abuse, medical trauma, motor vehicle accidents, and direct exposure to acts of violence such as homicides or suicides. Traumatic experiences are an important public health concern owing to their high prevalence, with 30–40% of adults reporting significant adverse experiences during their early life according to the literature.^28^ The differences between traumatic events and traumatic experiences have been elaborated in the scientific literature and in psychiatric disorder classification systems such as the DSM-5 TR and ICD-11. According to these classifications, traumatic experiences include repeated exposure to physical and/or emotional neglect, maltreatment and sexual abuse. Although these exposures may not match the intensity of singular traumatic events, they nonetheless predispose individuals to syndromes such as complex PTSD (C-PTSD). Recent research indicates a convergence between the concepts of ACEs – representing concrete situations of social, personal, and interpersonal distress that negatively affect a child's emotional development – and ELS and ELT; depending on their operational definitions and contextual factors, these experiences exhibit significant overlap, demonstrating the complex interplay between different forms of early life adversity and their potential to precipitate enduring psychological effects.^29,30^ ELT encompasses a range of experiences potentially harmful to those under 18 years old.^31^ Epidemiological research has demonstrated a strong link between ELT and mental health issues throughout life,^32^ indicating that such traumas cause lasting changes to the hypothalamic–pituitary–adrenal (HPA) axis and affect neurophysiological and inflammatory responses.^33^ Defined as any negative childhood experience with long-term harm potential (Table 1), these events can disrupt crucial developmental stages, affecting a child's emotional regulation and relationship-building.^40^ Trauma includes abuse, neglect, exposure to violence, and life upheavals such as parental divorce or unstable living situations, which may be further complicated by domestic violence or separation from caregivers.^39^ Early traumas undermine a child's sense of security and can have profound effects on development and health. They also set the stage for stress-related disorders by altering neurodevelopment and mental health. Neurobiologically, trauma can disrupt stress response systems and reducing hippocampal volume, which is essential for stress management and memory.^38^ Genetic factors and gene–environment interactions, such as *FKBP5* polymorphisms, can increase vulnerability to stress and affect cortisol regulation.^13^ Epigenetic mechanisms such as DNA methylation play an equally important part in modulation of gene expression without altering the DNA sequence.^45^ DNA methylation is a process in which a methyl group is added to specific DNA bases, often resulting in decreased expression of the corresponding gene. In response to trauma or stress, changes may occur in the methylation of specific genes involved in the stress response, such as the *NR3C1* gene that codes for the glucocorticoid receptor.^46^ However, not all individuals who experience childhood trauma develop psychopathological disorders,^42^ and the course and outcomes of early traumas can vary enormously from one individual to another, owing to many complex factors including genetics, environment, personal resources, individual resilience; social support can have a protective role, mitigating the negative effects of trauma.^42^ Childhood trauma is associated with a wide range of physical health conditions including cardiovascular diseases, immune system dysfunctions, endocrine disorders, gastrointestinal issues, chronic pain and respiratory diseases.^44^ The likelihood of developing specific mental health disorders, including depression, anxiety disorders, eating disorders, dissociative disorders, sleep disorders, attention-deficit hyperactivity disorder (ADHD), PTSD, C-PTSD, substance use disorders, and personality disorders, is influenced by childhood traumas.^28,34,47^ These disorders can be addressed through a combination of psychological and pharmacological interventions, depending on the individual needs of the patient. Psychological interventions may include cognitive–behavioural therapy (CBT), exposure therapy, mentalisation-based therapy and emotion regulation therapy, whereas pharmacological therapy can be used to treat specific symptoms such as anxiety or depression.^40,41^ Treatment of early trauma always requires an integrated and personalised approach, taking into account the individual needs of the patient and their specific traumatic experiences. ELT is associated with various comorbidities, although there has been a particular focus on the heightened risk of developing PTSD and its severe form, C-PTSD.^34,37,47,48^ Studies indicate that the prevalence of these disorders varies, with approximately 28% of adults who have experienced childhood abuse meeting the criteria for PTSD and 32% meeting those for C-PTSD.^35,36^ Whereas PTSD typically stems from a single traumatic event and is characterised by symptoms such as flashbacks and hyperarousal, C-PTSD results from prolonged traumas and involves issues with emotional regulation and attachment. In addition, the repercussions of early trauma extend beyond the individual, encompassing intergenerational effects that affect the mental health and well-being of future generations.^43^ Neuroendocrine dysregulation, which affects a range of physiological and psychological functions, has been identified as a factor contributing to the co-occurrence of depressive disorders and post-traumatic disorders.^49,50^

**Table 1:** Effects of early life trauma, including neurobiological, psychological, cognitive and social impacts, comorbidities and intergenerational transmission

Neurobiological impact	Early life trauma can result in enduring neurobiological changes, such as alterations in the hypothalamic–pituitary–adrenal axis, affecting stress response mechanisms.^33^
Psychological sequelae	The trauma may initially manifest as acute stress reactions in children, which can later evolve into chronic post-traumatic stress disorder, general anxiety disorders or other mood disorders.^34–36^
Comorbidity	Traumatic experiences in early life often set the stage for comorbid conditions such as depression, substance misuse or somatic symptom disorders, complicating diagnosis and treatment.^35–37^
Cognitive disruptions	Early trauma can also affect cognitive functions, leading to attention deficits, memory problems and impaired executive function that resemble symptoms of attention-deficit hyperactivity disorder.^13,38^
Social implications	The victim may experience difficulties in forming stable relationships or emotional attachments, or may even develop antisocial tendencies.^39^
Adaptive malfunctions	Trauma often affects the development of coping strategies, leading individuals to adopt maladaptive ways of dealing with stress, potentially contributing to the perpetuation of post-traumatic symptoms.^40,41^
Resilience and vulnerability factors	Individual genetic predispositions and environmental elements such as social support and early interventions can either mitigate or exacerbate the transition from early life trauma to adult post-traumatic disorders.^42^
Clinical complexity	Adults who experienced early life trauma may present with more severe symptoms, show a less favourable response to treatment and a more complex clinical picture, including ‘masked’ symptoms that can be mistakenly attributed to other conditions.^40,41^
Intergenerational transmission	There is evidence to suggest that untreated post-traumatic disorders can influence parenting behaviours, thereby affecting the next generation and potentially creating a cycle of trauma and disorder.^43^
Somatic health	Emerging evidence also indicates links between early life trauma and later development of chronic physical conditions such as cardiovascular disease or diabetes.^44^

Given the absence of systematic reviews thoroughly exploring the interplay between ELT and DTD, we considered that a scoping review specifically focused on this subject would be timely and relevant. In this review, we aimed to catalogue and analyse the body of existing studies, highlighting both well-established areas of research and those that are emerging or insufficiently explored. Moreover, we endeavoured to provide an updated compendium to outline both current knowledge and information gaps and guide future research. Overall, the goal was to broaden our understanding of the impacts of ELT on DTD and to facilitate the development of targeted and effective therapeutic approaches based on robust and relevant evidence.

## Materials and method

### Objectives

This scoping review aimed to identify and explore the scientific literature on the role of ELT and its outcomes in DTD. The review was performed in line with the PRISMA Extension for Scoping Review criteria.^51^

### Method

**Fig. 2:**
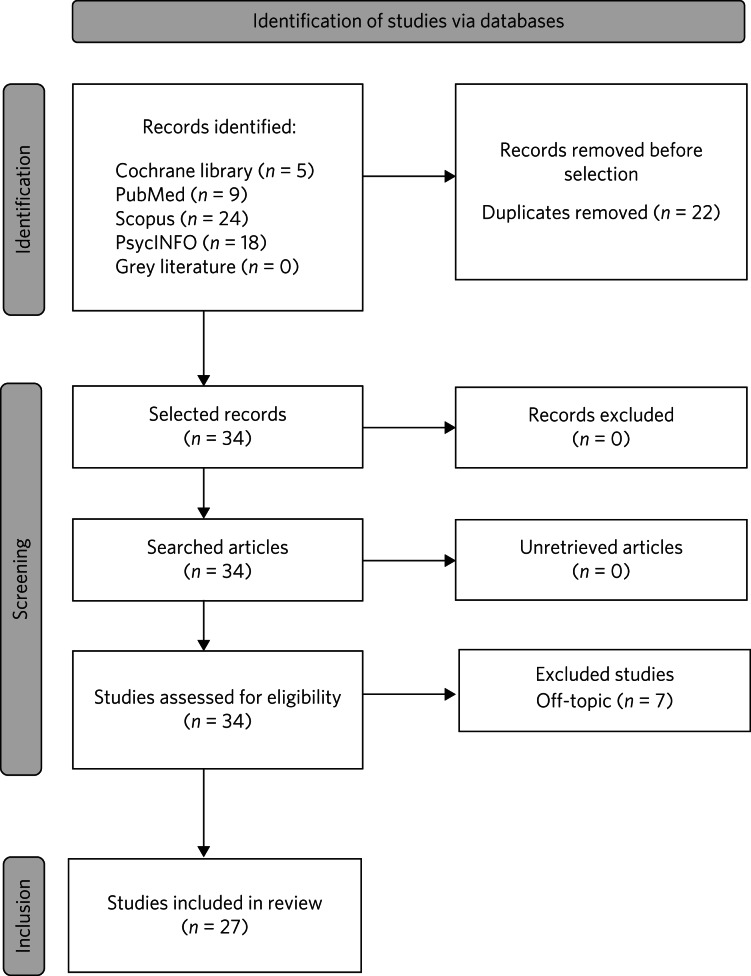
PRISMA flow chart.

We conducted a thorough literature search across multiple databases, including the Cochrane Library, PubMed, Scopus and PsycINFO, as well as searching for grey literature in OPEN GREY, using the following keyword string: (‘early life trauma’ OR ‘childhood trauma’ OR ‘early adversity’) AND (‘difficult-to-treat depression’ OR ‘treatment-resistant depression’ OR ‘refractory depression’) AND (‘longitudinal’ OR ‘outcome’ OR ‘follow-up’). The search parameters were tailored to the respective fields of each database. This search yielded 56 research articles from 1 January 2013, to 21 October 2023 (Fig. 2).

#### Study selection

Initially, the retrieved citations were independently screened by two blinded reviewers (W.P. and S.S.) at the title and/or abstract level, with discrepancies resolved by reaching a consensus. Potentially relevant reports were then fully assessed based on explicit selection criteria. Inclusion criteria included: (a) human studies; (b) studies involving patients with treatment-resistant or difficult-to-treat depressive disorders, particularly those that characterised and explained these conditions in the context of childhood trauma; and (c) studies of interventions intended to treat childhood traumas in the context of DTD. Exclusion criteria were: (a) non-human contexts; (b) report duplications, (c) off-topic research and (d) studies focusing on combat-related traumas.

#### Data extraction

Data were independently extracted by two blinded reviewers (W.P. and S.S.) using predefined electronic forms, with disagreements resolved by reaching a consensus. The end-points of interest for this scoping review were the relationship between early childhood trauma and treatment-resistant or difficult-to-treat depressive disorders in adults. Given the exploratory yet comprehensive scope of this review, no explicit primary end-point was specified.

#### Internal validity and quality assessment

The quality of the included studies was independently assessed by two blinded reviewers (W.P. and S.S.). The quality assessment tools used were the Jadad scale for randomised controlled trials, the STROBE checklist for observational studies and the AMSTAR checklist for meta-analyses. For survey studies, letters and reviews, qualitative assessment was performed.

#### Synthesis of results

The extracted data were thematically synthesised to identify common patterns and key themes across the studies. This involved grouping studies by their primary focus (for instance, intervention studies, epidemiological studies and reviews), identifying key findings and findings related to the impact of early trauma on DTD, and summarising the findings using tables and figures to highlight significant patterns and themes. For details of the assessment criteria and results, see Table 2.

**Table 2:** Summary of studies

Study	Study design	Quality assessment tool	Score/rating	Bias identified
Tunnard et al, 2014^52^	Cross-sectional study	STROBE checklist	High quality	Selection bias
Brakemeier et al, 2015^53^	RCT	Jadad scale	4/5	Selection bias
Cladder-Micus et al, 2015^54^	RCT	Jadad scale	4/5	Selection bias
Stevenson et al, 2016^55^	Preliminary study	Qualitative assessment	Relevant	Selection bias, recall bias
Michalak et al, 2016^56^	RCT	Jadad scale	4/5	Selection bias
Nelson et al, 2017^57^	Meta-analysis	AMSTAR	High quality	Publication bias
Minelli et al, 2019^58^	RCT	Jadad scale	4/5	Selection bias
O'Brien et al, 2019^59^	Prospective cohort study	STROBE checklist	High quality	Selection bias, recall bias
Li et al, 2019^60^	Survey	Qualitative assessment	Relevant	Selection bias
Robakis et al, 2019^61^	RCT	Jadad scale	3/5	Selection bias
Strawbridge et al, 2019^62^	Longitudinal study	STROBE checklist	High quality	Selection bias
Nikkheslat et al, 2020^63^	Cross-sectional study	STROBE checklist	High quality	Selection bias
Yrondi et al, 2020^64^	Cohort study	Qualitative assessment	High quality	Selection bias, recall bias
Gruhn et al, 2021^65^	RCT	Jadad scale	4/5	Selection bias
O'Brien et al, 2021^66^	RCT	Jadad scale	4/5	Selection bias
Yrondi et al, 2021^67^	Observational study	Qualitative assessment	High quality	Selection bias, recall bias
Taylor et al, 2021^68^	Longitudinal study	STROBE checklist	High quality	Selection bias
Fischer et al, 2021^69^	Observational study	STROBE checklist	High quality	Selection bias, recall bias
Levy et al, 2021^70^	Observational study	STROBE checklist	High quality	Selection bias
O'Brien et al, 2023^71^	RCT	Jadad scale	4/5	Selection bias
Maruani et al, 2023^72^	Prospective cohort study	Qualitative assessment	High quality	Selection bias, recall bias
Lichter et al, 2023^73^	Prospective observational cohort and case–control protocol	Qualitative assessment	High quality	Selection bias
Cladder-Micus et al, 2023^74^	Cohort study	Qualitative assessment	High quality	Selection bias
Giampetruzzi et al, 2023^75^	Cross-sectional study	STROBE checklist	High quality	Selection bias, recall bias
Fond et al, 2023^76^	Prospective cohort study	Qualitative assessment	High quality	Selection bias, recall bias
Benjamin et al, 2023^77^	RCT	Jadad scale	3/5	Selection bias
Kuzminskaite et al, 2023^78^	Meta-analysis	AMSTAR	High quality	Publication bias

RCT, randomised controlled trial.

### Results

This scoping review of the scientific literature found that there have been limited numbers of studies explicitly investigating the role of ELT in DTD. This may be because the DTD paradigm is still in its early stages, and therefore no substantial body of research on its correlation with ELT has yet been generated. However, studies have explored the mechanisms through which ELT may affect depressive conditions that are related to the emerging concept of DTD. The studies reviewed here varied widely in their methodologies, populations and definitions of ELT and DTD, complicating the synthesis of findings and the potential to draw definitive conclusions. We detected potential biases in the studies, including selection bias and recall bias, which could affect the validity of the results. We also noted a paucity of longitudinal studies that could provide insights into the long-term effects of ELT on DTD; such studies will be crucial for understanding the progression and treatment of the condition. One study examined phenotypic data from the 23andMe survey and identified a significant correlation between experience of traumatic life events, especially in childhood, and severity of TRD.^60^ However, the precise mechanisms underlying this relationship require further investigation. In the field of psychotherapy, the cognitive–behavioural analysis system of psychotherapy (CBASP) is a model particularly relevant to treatment of depression due to childhood trauma. CBASP incorporates elements of CBT and psychodynamic psychotherapy and is designed to help patients understand the impact of their behaviour on others and modify dysfunctional behavioural patterns through a process called ‘personal engagement discipline’. However, Brakemeier et al^53^ found that the integration of CBASP with standard pharmacological therapies could initially exacerbate depression symptoms, particularly when addressing issues of trauma in the initial stages of therapy. Concomitantly, Stevenson et al^55^ reported substantial improvements in patients with TRD and histories of childhood trauma using the conversational model, despite the presence of comorbid severe personality disorders. Customisation is another key aspect of treatment for patients with TRD. Results of the GEParD and DaCFail studies (research projects exploring various facets of TRD) suggest that personalisation based on variables such as comorbidity, genetic profiles, and history of traumatic life events is a promising approach to developing frameworks to treat DTD.^73^ However, despite extensive research, the precise role to which childhood trauma needs to be taken into account in treatment approaches remains unclear. Taylor et al^68^ found that ELT had predictive value for TRD in hospital settings, indicating a need for personalised therapeutic strategies. Strawbridge et al^62^ investigated the biological links between traumatic experiences and neuroinflammation in patients with TRD and identified a connection between ELT and elevated IL-12p70 levels. Maruani et al^72^ explored the relationships among sleep patterns, depressive symptoms and childhood trauma and found that evening chronotype may exacerbate depression. However, conflicting findings were reported by the Childhood Trauma Meta-Analysis Study Group, which found that MDD patients with ELT responded well to both pharmacological and psychotherapeutic treatments. Benjamin et al^77^ found that sexual and gender minority (SGM) patients with TRD often had more severe histories of childhood trauma and increased suicide risk. However, treatment recommendations did not significantly differ between SGM and non-SGM groups, suggesting the need for more tailored approaches. Gruhn et al^65^ emphasised the complex responses of ELT patients to pharmacological and CBT and advocated the personalisation of treatment. Tunnard et al^52^ reported that childhood trauma may lead to a more severe course of depressive illness and increased suicide risk in patients with TRD. In 2015, Cladder-Micus et al^54^ suggested that mindfulness-based cognitive therapy (MBCT) could be effective for ELT patients; however, their 2023 follow-up did not confirm this finding.^74^ Fischer et al^69^ examined the potential correlations of childhood trauma, TRD and levels of high-sensitivity C-reactive protein, raising questions about biomarkers of childhood trauma. Minelli et al^58^ studied the effects of trauma-focused psychotherapies (eye movement desensitisation and reprocessing (EMDR) and trauma-focused CBT) in TRD patients with trauma histories and found that EMDR showed marginal superiority in reducing depressive symptoms. Fond et al^76^ investigated long-term benzodiazepine use by patients with TRD and reported potential links with childhood trauma. Yrondi et al^64^ found that childhood maltreatment was associated with increased TRD severity, indicating a need for more personalised therapies. They subsequently extended this research to a geriatric population,^67^ finding an association of childhood physical abuse with depression severity in advanced age. O'Brien et al^59,66,71^ suggested that ketamine may be effective for ELT patients with TRD, particularly those with histories of childhood abuse. Nelson and colleagues, in their 2017 meta-analyses, demonstrated a significantly higher risk of depression, especially severe depression and TRD, in individuals with histories of childhood maltreatment.^57^ In 2020, Nikkheslat et al^63^ reported decreased antidepressant efficacy in those with childhood traumas, potentially linked to hyperactivation of the HPA axis. Michalak et al^56^ and Stevenson et al^55^ explored the impact of childhood adversities on chronic depression through various therapeutic approaches. Michalak et al found MBCT and CBASP to be more effective for chronic depression, especially in patients with experience of childhood adversities, whereas Stevenson et al emphasised the role of trauma-informed psychodynamic psychotherapy in treating chronic TRD, which is often associated with personality disorders and early trauma. In summary, studies highlight the need for personalised, trauma-informed approaches to treating DTD in individuals with a history of ELT. For details of the reviewed studies, see Supplementary Table 1 available at https://doi.org/10.1192/bjb.2024.75.

Several recurring themes emerged from our review, which can be summarised as follows.

- Role of ELT: childhood traumas significantly affect the development of depression, its severity and resistance to conventional treatments.
- Underlying mechanisms: there is growing interest in mechanisms such as chronic inflammation that could underlie the connections between childhood traumas and DTD.
- Biomarkers: identifying biomarkers associated with childhood traumas could provide tools for more precise diagnosis and treatment.
- Comorbidities: medical, metabolic and psychiatric comorbidities are often correlated with a history of adversity in childhood and can complicate treatment responses.
- Complexity of treatment: traumas experienced in childhood can affect the efficacy of therapies, suggesting the need for a personalised therapeutic approach.
- Overcoming traditional limits: as individuals with ELT may experience reduced effectiveness of antidepressants, there is a need for personalised and innovative treatment approaches such as CBASP and the conversational model, although more research is required to confirm their efficacy in patients with ELT; ketamine is also being explored with respect to its potential use in treatment-resistant cases.
- Trauma-focused psychotherapies: the efficacy of therapies such as EMDR and trauma-focused CBT in patients with DTD is an emerging area of research.
- Methodological limitations: further research with larger samples and more rigorous methodologies is needed to confirm the findings of most of the studies.

ELT has a significant impact on the pathogenesis and treatment of DTD is significant, although gaps in knowledge persist. As well as increasing the risk of DTD, early traumas may complicate its management, reducing the effectiveness of standard therapies. Given the enduring effects of ELT, the importance of personalised treatments must be emphasised. A comprehensive evaluation of childhood trauma history is essential for effective management of DTD. An integrated clinical approach, encompassing pharmacological, psychotherapeutic and neurostimulatory therapies tailored to individual cases, is recommended. Further research is warranted to refine therapeutic strategies for this specific patient subgroup.

## Discussion

**Fig. 3:**
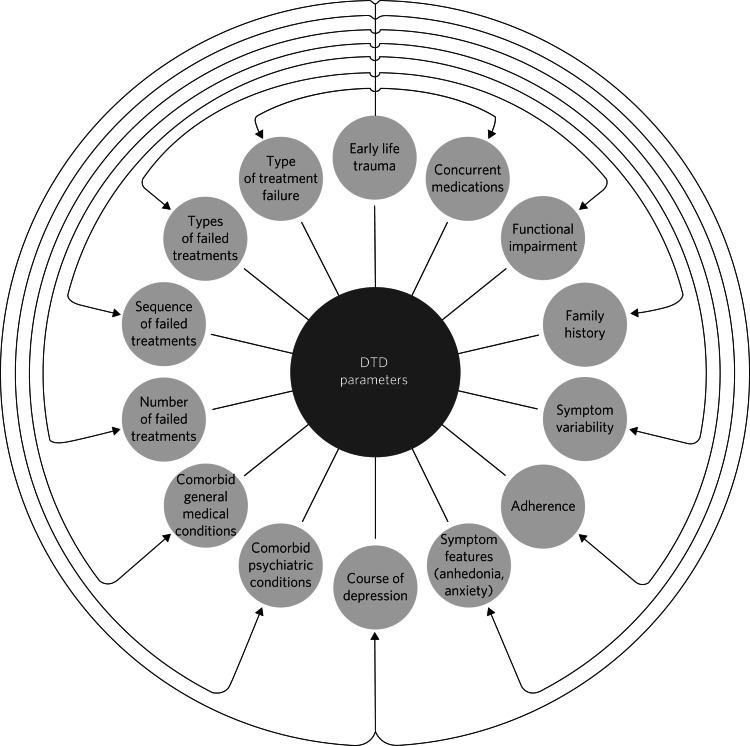
The relationship between early life trauma (ELT) and difficult-to-treat depression (DTD), in which ELT is a significant risk factor, not only contributes to the development and persistence of depressive disorders but also complicates their clinical management.

The results of our scoping review indicate a complex relationship between ELT and DTD, in which ELT is a significant risk factor that not only contributes to the development and persistence of depressive disorders but also complicates their clinical management. The impact of ELT on various parameters of DTD (Fig. 3) indicates how early adversities affect multiple dimensions of an individual's life, including overall resilience, treatment response and functional outcomes, and have a crucial role in shaping the parameters that contribute to the manifestation of DTD.

ELT is associated with elevated rates of treatment failure, with individuals often experiencing multiple failed attempts owing to the complex interaction between trauma-induced neurobiological changes and psychological adaptations. These factors reduce the effectiveness of standard pharmacological and psychotherapeutic interventions. The chronicity and severity of depressive symptoms in patients with ELT mean that they may receive various forms of therapy, not all of which are effective, resulting in a sequence of failed treatments. Thus, there is a need for personalised therapeutic strategies. ELT significantly contributes to the development of both psychiatric and medical comorbidities, including PTSD, anxiety disorders, personality disorders, substance use disorders and chronic pain syndromes, further complicating the clinical picture of DTD and making remission difficult to achieve.

Patients with DTD often have significant functional impairments in various areas of life, including interpersonal relationships, occupational functioning and overall quality of life. The variability in symptom presentation further complicates the course and treatment of their depression. The presence of ELT interacts with genetic predispositions, influencing familial patterns of depressive disorders and suggesting that individuals with a family history of depression and ELT are at higher risk of developing more severe and treatment-resistant forms of depression. Neurobiological alterations resulting from ELT, such as changes in the HPA axis and increased levels of inflammatory markers, contribute to the pathophysiology of DTD, affecting the brain's stress response systems and reducing the efficacy of antidepressants and other therapeutic interventions. ELT is closely linked to specific symptomatic features such as anhedonia and anxiety, which are difficult to treat. The course of depression in these patients is often severe and chronic, with frequent relapses and incomplete remissions.^52,57,63,64^

Trauma-related factors can also influence treatment adherence, as individuals with ELT may have trust issues and difficulty establishing therapeutic alliances. In addition, concomitant medications for comorbid conditions can further complicate the management of DTD. When DTD is suspected, clinical re-evaluations are strongly recommended to detect possible ELT as an underlying cause of DTD. The presence of such disorders can lead to treatment resistance, facilitated by the negative mutual interference of the two disorders and the individual's poor coping and problem-solving skills, as well as a lack of general resilience.^75^

The presence of ELT in the anamnesis is associated with an increased risk of relapse in depressive disorders, often triggering a persistent and recurrent course of the illness, and there is evidence of more psychopathological manifestations such as anxiety, anhedonia and apathy in such cases.^74^ The emotional consequences of trauma can also translate into increased suicidal ideation and profound emotional distress, culminating in a pervasive sense of despair.^70^ This in turn affects emotional, cognitive and somatic domains, leading to complex interactions between ELT and depressive symptoms in adulthood.^61^ The difficulty of treatment is further exacerbated by neurobiological alterations resulting from childhood trauma, which can lead to impulsivity and high-risk behaviours that impede adherence to care.^63,69^ Hyperarousal states resulting from severe traumas, which are characterised by hypervigilance and impaired concentration, can disrupt attentional processes, generating symptoms similar to those of ADHD;^79^ indeed, these states can be mistaken for ADHD. In the therapeutic context, those who do not respond or only partially respond to treatments often have more severe trauma histories compared with those who show a favourable response, indicating a negative impact of childhood adversity on treatment outcomes. Over time, the cumulative effects of childhood trauma can lead to marked functional decline and increased symptom variability. Therefore, early identification of childhood trauma is crucial for the effective management of DTD, as is the use of a targeted and personalised therapeutic approach.

Integration of evidence-based therapies such as EMDR and trauma-focused CBT is fundamental to directly addressing the role of ELT in DTD. EMDR, in which eye movements are used to help patients process and overcome the emotional effects of traumatic memories, is particularly effective in treating PTSD. Similarly, trauma-focused CBT helps patients to restructure negative thoughts related to trauma, providing effective strategies for managing emotions and reactions. Integrating these therapies into the treatment plan for TRD could offer more effective care pathways, overcoming the barriers to healing imposed by trauma.^56,58,74^

Overall, approaches based on the use of psychotherapies in conjunction with pharmacological therapy have proven particularly effective for patients with DTD.^54,55,65,78^ ELT not only increases the risk of developing DTD but is also closely linked to the severity of symptoms and less favourable treatment outcomes.^52,57,64,67^ This results in more severe symptoms, resistance to standard therapies and ‘masked’ disorders that can be misinterpreted as other conditions. The comorbidity between PTSD and DTD adds a further layer of complexity, negatively influencing the patient's social interactions and coping strategies. Directly addressing the role of childhood trauma can be decisive in improving the treatment response of patients with depression. A comprehensive assessment of the presence of ELT is necessary, both to identify the full clinical expression of the patient's psychiatric disorder and evaluate their prognosis, and to develop an effective and exhaustive care pathway that considers the relative importance of all aspects of the disorder (psychopathological, cognitive, global functioning, psycho-affective).

Our review, despite intrinsic limitations owing to the scarcity of scientific literature on the topic, unequivocally confirms the close connection between childhood trauma in relation to TRD. This indicates that psychotherapeutic interventions should be considered to be an integral part of the treatment of DTD.

## Conclusions

This review underscores several pivotal point, which can be summarised as follows.

- The established correlation between ELT and DTD is fundamental to both our understanding of DTD and its therapeutic management. Recognising this relationship will be vital in tailoring treatment approaches that directly address the underlying trauma, potentially leading to improved clinical outcomes.
- Management of DTD requires integrated and tailored therapies specifically targeting the repercussions of ELT. Personalised treatment plans are key; these should aim not only to improve DTD symptoms but also to provide patients with the means to overcome the multiple psychological and health challenges resulting from early childhood adversity. Recognising and addressing childhood trauma is central to improving therapeutic outcomes for patients with DTD, and there is a need to adopt psychotherapies specifically directed at these intertwined conditions.
- There is an urgent need to advance our understanding of DTD and develop innovative diagnostic and therapeutic strategies that acknowledge the impact of childhood trauma. This will require methodologically robust studies with ample sample sizes to validate and extend existing knowledge. Such research endeavours should investigate the neurobiological and psychosocial mechanisms linking ELT to DTD, with the aim of guiding the development of more effective treatments for this complex condition.

Addressing the nuanced interplay between ELT and DTD is of paramount importance. A multidisciplinary approach based on comprehensive research and innovative treatments will be essential to overcoming the challenges of DTD and offering hope and improved care for those affected.

## Supporting information

Paganin et al. supplementary material Paganin et al. supplementary material

## Data Availability

The data that support the findings of this study are available from the corresponding author, W.P., upon reasonable request.

## About the authors

**Walter Paganin** is a psychiatrist-psychotherapist and PhD candidate in neuroscience at the University of Tor Vergata, Rome, Italy. **Sabrina Signorini** is a psychologist and psychotherapist at the StudioPsicologiaSignorini, Guidonia, Rome, Italy. **Antonio Sciarretta** is a psychiatrist at IHG, Guidonia, Rome, Italy.
