# Supplementary material for: Bridging early life trauma to difficult-to-treat depression: scoping review
Source: BJPsych Bull. 2025 Dec;49(6):412–23. doi: 10.1192/bjb.2024.75 (PMC12676230; doi:10.1192/bjb.2024.75)
Supplement: Paganin et al. supplementary material [file S2056469424000755sup001.docx]

| **Study and Authors** | **Study Partecipants** | **Intervention** | **Detailed Abstract Summary** | **Outcomes Measured** | **Author Conclusions** |
| --- | --- | --- | --- | --- | --- |
| The impact of childhood adversity on suicidality and clinical course in treatment-resistant depression.  (Tunnard et al., 2014) | 137 patients with TRD | Associations between childhood adversity, depressive symptoms and clinical course were investigated. | This study investigated the impact of childhood adversity on suicidality and clinical course in treatment-resistant depression. It found that childhood adversity was associated with poorer clinical course, including earlier age of onset, episode persistence and recurrence, and predicted lifetime suicide attempts and psychosis. | •Clinical course.  •Age of onset.  •Episode persistence.  •Recurrence.  •Lifetime suicide attempts.  •Lifetime psychosis. | Childhood adversity is related to a more severe clinical course and a higher incidence of suicide attempts and psychosis. |
| Overcoming treatment resistance in chronic depression: A Pilot Study on outcome and feasibility of the Cognitive Behavioral Analysis System of psychotherapy as an inpatient treatment program.  (Brakemeier et al., 2015) | 20 treatment resistant inpatients with treatment resistant chronic depression. | Cognitive Behavioral Analysis System of Psychotherapy (CBASP). | This study investigated the feasibility and outcome of the Cognitive Behavioral Analysis System of Psychotherapy (CBASP) as an inpatient treatment program for treatment-resistant chronic depression. It found that 75.0% of patients sustained response after 6 months and 48.0% after 12 months, but that continuation of treatment after discharge should be optimized. | •Remission.  •Response.  •Temporary deterioration of symptoms during treatment. | CBASP is feasible and effective for chronic treatment-resistant depression, but continuation treatment should be optimized. |
| A randomized controlled trial of Mindfulness-Based Cognitive Therapy (MBCT) versus treatment-as-usual (TAU) for chronic, treatment-resistant depression: study protocol.  (Cladder-Micus et al., 2015) | Not specified. | Mindfulness Based Cognitive Therapy (MBCT). | This study is conducted to compare Mindfulness-Based Cognitive Therapy (MBCT) with treatment-as-usual (TAU) for chronic, treatment-resistant depression. The primary outcome is depressive symptoms, with secondary outcomes including remission rates, quality of life, rumination, mindfulness skills and self compassion. | •Depressive symptoms.  •Remission rates.  •Quality of life.  •Rumination.  •Mindfulness skills.  •Self Compassion. | MBCT is effective in reducing depressive symptoms and improving quality of life and mindfulness skills in treatment-resistant depression. |
| Trauma, personality disorders and chronic depression -the role of the conversational model of psychodynamic psychotherapy in treatment resistant depression.  (Stevenson et al., 2016) | 44 patients (13 males and 31 females) with TRD, comorbid personality disorders, and histories of early childhood trauma. | Conversational Model (CM) of psychodynamic psychotherapy. | This study studied the interim treatment outcome of 44 patients with treatment-resistant depression. It found that patients responded with symptomatic and functional improvement to twice weekly Conversational Model psychotherapy. | •Symptoms.  •Functioning.  •Self Esteem.  •History of trauma.  •Personality functioning.  •Suicidality. | The Conversational Model of psychotherapy leads to symptomatic and functional improvements in patients with TRD. |
| Mindfulness-Based Cognitive Therapy and a Group.Version of the Cognitive Behavioral Analysis System of Psychotherapy for Chronic Depression: Follow-Up Data of a Randomized Controlled Trial and the Moderating Role of Childhood Adversities.  (Michalak et al., 2016) | 71 participants chronic depressed. | CBASP and MBCT. | The Research Ethics Committee of the German Psychological Association approved the study. | •24-item Hamilton Depression Rating Scale (HAM-D).  •Beck Depression Inventory, second edition (BDI-II).  •Childhood Trauma Questionnaire. | MBCT and CBASP are effective in treating chronic treatment-resistant depression, with MBCT moderated by childhood adversities. |
| Childhood-maltreatment-and-characteristics-of-adult-depression-meta-analysis.  (Nelson et al., 2017) | Not specified. | Not specified | This meta-analysis finds that childhood maltreatment is a risk factor for severe, early-onset, treatment-resistant depression. | •Severity of depression  •Age of onset of depression.  •Treatment resistance of depression.  •Course of depression. | Childhood maltreatment is associated with greater severity, earlier onset, and treatment resistance in depression. |
| Clinical efficacy of trauma-focused psychotherapies in treatment-resistant depression (TRD) in-patients: A randomized, controlled pilot-study.  (Minelli et al., 2019) | 22 treatment resistant patients. | Trauma focused cognitive behavioural therapy (TF CBT) and/or eye movement desensitization and reprocessing (EMDR). | This study investigated the efficacy of trauma-focused psychotherapies in treatment-resistant depression (TRD) patients. It found that EMDR was more effective than TF-CBT in reducing depressive symptomatology and that this effect was partly maintained at 24 weeks. | •Depressive symptom remission.  •Depressive symptom scores. | EMDR is effective in reducing depressive symptoms in patients with TRD. |
| The impact of childhood maltreatment on intravenous Ketamine Outcomes for adult patients with Treatment-Resistant Depression.  (O’Brien et al., 2019) | 115 for a single infusion and 63 for a course of repeated infusions. | Intravenous ketamine | This study examines the relationship between childhood maltreatment and treatment response to intravenous ketamine in patients with treatment-resistant depression. It finds that clinically significant childhood maltreatment is associated with better treatment response to a single and repeated infusions and higher maltreatment load is associated with a higher remission rate. | •Quick Inventory of Depressive Symptoms Self Report (QidsSr). | Childhood maltreatment may improve the response to ketamine treatment in patients with TRD. |
| Phenotypic analysis of 23andMe survey data: Treatment-Resistant Depression from participants’ perspective.  (Li et al., 2019) | Approximately 56,000 individuals with depression. | This survey identified depression characteristics, comorbidities, trigger events, and early childhood trauma that distinguish TRD from NTRD. | 3409 participants with self-reported TRD tended to have younger age of onset, and a more persistent course prior to initiation of treatment than the 18,511 participants classified as NTRD. | •Effect of pharmacotherapy during the depressive episode.  •Non response to at least 2 antidepressants taken for at least 5-6 weeks.  •Responsive to either the 1^st^ or 2^nd^ medication taken for at least 3-4 weeks.  •Depression characteristics.  •Comorbidities.  •Trigger events.  •Early Childhood Trauma. | Participants with TRD had a younger age of onset and a more persistent course compared to participants with NTRD. |
| Early life adversity blunts responses to pioglitazone in depressed, overweight adultes.  (Robakis et al., 2019) | Not specified. | Pioglitazone for augmentation of standard treatments for depression. | This experimental study examines the effects of early life adversity on the metabolic response to pioglitazone. It finds that early life adversity significantly impairs the metabolic response to pioglitazone and may contribute to resistant depression via the generation of an insulin resistant phenotype. | •Metabolic response to Pioglitazone.  •Changes in depressive symptoms. | Early life adversity impairs the response to pioglitazone therapy in overweight depressed patients. |
| Inflammatory profiles of severe treatment-resistant depression.  (Strawbridge et al., 2019) | 36 patients with treatment resistant depression and a matched sample of non depressed controls. | Specialist inpatient treatment program. | Elevated interleukins 6 and 8 were associated with poorer treatment outcomes in treatment-resistant depression patients. | •27 Inflammatory proteins.  •Depression severity scores before and after admission.  •Treatment outcomes. | Inflammatory proteins are correlated with poorer treatment outcomes in TRD. |
| Childhood trauma, HPA axis activity and antidepressant response in patients with depression  (Nikkheslat et al., 2020) | 218 people, with 163 depressed patients and 55 healthy volunteers. | Antidepressant medication. | This study investigated the link between childhood trauma, HPA axis activity and antidepressant response in patients with depression. It found that treatment non-responder patients had higher exposure to childhood trauma and the severity of childhood trauma was associated with increased diurnal cortisol levels only in individuals with glucocorticoid resistance. | •Childhood Trauma Questionnaire Score.  •17-Item Hamilton Rating Scale for Depression Score.  •Baseline serum C reactive protein level.  •Salivary cortisol level at multiple time points during the day.  •Presence of glucocorticoid resistance. | Exposure to childhood trauma is associated with higher resistance to antidepressant treatments. |
| Childhood maltreatment and clinical severity of treatment-resistant depression in a French cohort of outpatients (FACE-DR): One-year follow-up.  (Yrondi et al., 2020) | Outpatients with treatment resistant depression (TRD) in a French cohort. | Not specified. | This study investigates the association between childhood maltreatment and treatment-resistant depression in a French cohort of outpatients. It finds that childhood maltreatment is associated with a higher risk of TRD and a more severe course of the disorder. | •Clinical severity of Treatment Resistant Depression. | Childhood maltreatment is associated with a higher risk and greater severity of TRD. |
| Early Life Adversity as a moderator of symptom change following Selective Serotonin Reuptake Inhibitors and Cognitive Behavioral Therapy.  (Gruhn et al., 2021) | 96 patients with depression. | Selective serotonin reuptake inhibitors (SSRIs) and cognitive behavioral therapy (CBT). | This study investigated the efficacy of SSRIs and CBT for individuals with and without Early Life Adversity (ELA). It found that individuals with a positive history of ELA had a greater reduction in depression symptoms following SSRIs compared with CBT, while individuals with a negative history of ELA had a greater reduction in depression symptoms following CBT compared with SSRIs. | •Internalizing disorder symptoms.  •Depression symptoms. •Anxiety symptoms. | SSRIs are particularly effective for individuals with ELA, while CBT is more effective for those without ELA. |
| Distinct trajectories of antidepressant response to intravenous ketamine.  (O’Brien et al., 2021) | 328 depressed adult outpatients referred to a community clinic. | Intravenous ketamine. | This study examined the response trajectories to intravenous ketamine in 328 depressed adult outpatients. It found three distinct response trajectories, with higher childhood physical abuse in the group with robust ketamine response. | •Depression severity assessed by the Quick Inventory of Depressive Symptomatology Self Report (Qids Sr) at baseline and before each infusion. | Childhood physical abuse is associated with a more robust response to ketamine. |
| Relationship between childhood physical abuse and clinical severity of treatment resistant depression in a geriatric population.  (Yrondi et al., 2021) | 96 treatment resistant patients. | Not specified. | This study investigated the relationship between childhood physical abuse and clinical severity of treatment-resistant depression in a geriatric population. It found a positive correlation between childhood trauma and depressive symptom severity, and a statistically significant correlation between physical abuse and depressive symptom severity. | •Montgomery and Asberg Depression Rating Scale (Madrs) mean scores.  •Quick Inventory Depression Scale Self Report (Qids Sr) mean scores.  •Self Esteem Scores. | Childhood trauma is associated with greater severity of symptoms in the geriatric population with TRD. |
| Predicting clinical outcome to specialist multimodal inpatient treatment in patients with treatment resistant depression.  (Taylor et al., 2021) | 174 patients at the Affective Disorders Unit of the Bethlam Royal Hospital with current depression and established TRD. | Specialist inpatient treatment program. | The study examines factors influencing treatment outcomes in treatment-resistant depression. Age at onset and episode count positively impact response, while treatment resistance is a negative predictor. Individual prediction remains uncertain. | •Response to treatment (50% Reduction in Hamd-21 Score at discharge). | Age at onset and number of episodes influence response to treatment of depression. |
| Assessing the links between childhood trauma, C-reactive protein and response to antidepressant treatment in patients with affective disorders  (Fischer et al., 2021) | 76 patients with uni or bipolar depression and 53 healthy controls. | Antidepressant treatment | This study explored the associations between Adverse Childhood Experiences (ACE), C-reactive Protein (CRP) levels, and response to antidepressant treatment in uni and bipolar depression. It found that sexual abuse was associated with higher CRP levels and emotional abuse with lower levels, but ACE and CRP did not show any significant associations with treatment response in the unipolar depressed subgroup. | •Depressive symptoms assessed by the Montgomery Asberg Depression Rating Scale (Madrs).  •Adverse Childhood Experiences (Ace) assessed by the Childhood Trauma Questionnaire (Ctq).  •Body Mass Index (Bmi).  •High sensitive C Reactive Protein (Hscrp) levels. | Childhood trauma influences inflammation levels but does not significantly associate with treatment response. |
| Occurrence of Side Effects in Treatment-Resistant Depression: role of clinical, socio-demographic and environmental characteristics.  (Levy et al., 2021) | 108 patients with treatment resistant depression in France. | Antidepressant treatment. | This observational study investigated the parameters associated with the occurrence of side effects in treatment-resistant depression. It found that age, sex, intensity of anxious, depressive and suicidal symptoms, and history of childhood trauma were associated with the occurrence of side effects. | •Overall occurrence of Side Effects.  •Profile of the Side Effects (9 Categories, 32 Items). | Various clinical and demographic factors are associated with side effects in TRD patients. |
| Replication of distinct trajectories of antidepressant response to intravenous ketamine.  (O’Brien et al., 2023) | 298 patients with major depressive disorder. | Intravenous ketamine infusions | This study investigated the response pathways associated with repeated intravenous ketamine infusions among patients with major depressive disorder. It found that only about a third of depressed patients benefitted substantially from the treatment. | •Depression (measured by Quick Inventory of Depressive Symptomatology Self Report (Qids Sr).  •Suicidality (measured by severity at entry and change in severity during treatment). | Ketamine is effective for about a third of MDD patients. |
| Diurnal symptoms of sleepiness and dysfunction predict future suicidal ideation in a French cohort of outpatients (FACE-DR) with treatment resistant depression: A 1-year prospective study about sleep markers.  (Maruani et al., 2023) | No mention found of participant count. Patients with treatment resistant depression (TRD) in France. | Mood, sleep and circadian rhythms were assessed at baseline; suicidal risk was assessed both at baseline and during a one-year follow-up with standardized subjective questionnaires | The study links sleep and circadian issues to suicidal ideation in treatment-resistant depression patients. Excessive daytime sleepiness raises suicide risk, while hypnotic use lowers it. | •Suicidal Thoughts. •Suicidal Ideation. | Sleep disturbances are associated with increased suicide risk in TRD patients. |
| Deep phenotyping as a contribution to personalized depression therapy: the GEParD and DaCFail protocols  (Lichter et al., 2023) | 346 depressed patients. | Antidepressant therapy. | Multimodal biomarkers including cardiovascular and (epi-)genetic markers, functional brain and heart imaging when evaluating the response to antidepressive therapy using comprehensive psychometry are useful in evaluating the response to antidepressive therapy using comprehensive psychometry. | •Mood.  •Emotions.  •Self concept.  •Neurocognition  •Somatic function.  •Cardiovascular markers.  •(Epi )Genetic markers.  •Functional Brain Imaging.  •Heart Imaging.  •Response to antidepressive therapy. | Multimodal biomarkers are useful for personalizing antidepressant therapy. |
| Follow-up outcomes of Mindfulness-Based Cognitive Therapy (MBCT) for patients with chronic, treatment-resistant depression.  (Cladder-Micus et al., 2023) | 106 chronically and treatment resistant depressed outpatients. | Mindfulness based cognitive therapy (MBCT). | This experimental study investigated the long-term outcomes of Mindfulness-Based Cognitive Therapy (MBCT) for chronically, treatment-resistant depressed patients. | •Depressive symptoms.  •Remission rates.  •Quality Of Life.  •Rumination.  •Mindfulness Skills.  •Self Compassion. | MBCT improves symptoms and quality of life in chronic TRD. |
| The impact of adverse childhood experiences on adult depression severity and treatment outcomes.  (Giampetruzzi et al., 2023) | 454 adult patients with treatment resistant depression (TRD) and major depression or persistent depressive disorder. | Not specified. | This study examines the impact of adverse childhood experiences on adult depression severity and treatment outcomes. It finds that greater ACE exposure is associated with more severe symptomatology and treatment outcomes, and that the ACE subtypes of sexual trauma and violence uniquely predict a lifetime suicide attempt(s). | •Symptom severity.  •Treatment Outcomes.  •History of Lifetime suicide attempt(S). •Lifetime inpatient admission(S). | ACE exposure predicts greater symptom severity and poorer treatment outcomes. |
| Long-term benzodiazepine prescription in treatment-resistant depression: A national FACE-TRD prospective study.  (Fond et al., 2023) | No mention found of participant count. Patients with treatment resistant depression (TRD) in France. | Not specified. | This study investigates the prevalence of long-term benzodiazepine use in a nationwide population of patients with treatment-resistant depression, the rate of patients succeeding at withdrawing benzodiazepines at one year, and if persistent use is associated with poorer mental health outcomes. | Not specified. | Long-term benzodiazepine use is associated with poorer mental health outcomes in TRD. |
| Treatment efficacy and effectiveness in adults with major depressive disorder and childhood trauma history: a systematic review and meta-analysis.  (Kuzminskaite et al., 2023) | 6830 participants (age range 18–85 years, male and female individuals and specific ethnicity data unavailable). More than half (4268 [62%] of 6830) of patients with major depressive disorder reported a history of childhood trauma. | Psychotherapy and Antidepressant therapy. | Contrary to previous studies, we found evidence that the symptoms of patients with major depressive disorder and childhood trauma significantly improve after pharmacological and psychotherapeutic treatments, notwithstanding their higher severity of depressive symptoms. | •Symptom severity.  •Treatment Outcomes. | Pharmacological and psychotherapeutic treatments are effective for patients with major depressive disorder and childhood trauma. |
| Differences in clinical presentations of patients seeking care for treatment-resistant depression across sexual orientations and gender identities.  (Benjamin et al., 2023) | TRD 52 SGM and 202 non-SGM) patients. | Psychotherapy and Antidepressant therapy. | The study aims to understand the clinical characteristics and treatment recommendations for Sexual and/or Gender Minority (SGM) individuals as compared to non-SGM individuals in the context of Treatment-Resistant Depression (TRD). | •Treatment Outcome. | SGM individuals with TRD require personalized therapeutic approaches. |

**TABLE 3.** Characteristics and Outcomes of Studies.
